# Relative team-member exchange, affective organizational commitment and innovative behavior: The moderating role of team-member exchange differentiation

**DOI:** 10.3389/fpsyg.2022.948578

**Published:** 2022-07-22

**Authors:** Chao Chen, Xinmei Liu

**Affiliations:** ^1^Business School, Hohai University, Nanjing, China; ^2^School of Management, Xi’an Jiaotong University, Xi’an, China

**Keywords:** relative team-member exchange, team-member exchange differentiation, affective organizational commitment, innovative behavior, social comparison theory

## Abstract

Based upon social comparison theory, a multilevel moderated-mediation theoretical model was built up to explore the influence mechanism of relative team-member exchange (RTMX) on innovative behavior. We tested the proposed hypotheses using a sample of 260 individual members within 51 teams in a two-wave survey study. Controlling for team-member exchange (TMX), results showed that RTMX was positively related to innovative behavior, and the relationship above was mediated by affective organizational commitment. Moreover, team-level TMX differentiation played a moderating role in the mediated relationship between RTMX and innovative behavior through affective organizational commitment. This study also emphasizes the significance of conceptualizing TMX as concurrently implementing at multiple levels.

## Introduction

Innovative behavior (i.e., “A multi-stage process of problem recognition, generation of ideas or solutions, building support for ideas, and idea implementation”; see Pieterse et al., 2010, p. 610) has been theorized to be highly critical for the development of both individuals and teams (Ali et al., 2019; Imam et al., 2020). Especially in today’s fiercely competitive surroundings it is more significant to achieve advantage by engaging in innovative behavior. More scholars also call for more attention to innovative behavior due to its importance. Thus, it is important and necessary to explore the antecedents of innovative behavior. Social exchange relationships embedding in the whole innovative process have been robustly examined to make a significant influence on employees’ behaviors including innovative behavior (Saeed et al., 2018). However, compared to several vertical social exchange relationships (e.g., LMX; perceived organizational support), very few prior studies have attempted to cast light on horizontal social exchange relationships (Farmer et al., 2015). Because individual members have to cooperate with each other to fulfill the challenging and various team tasks (Bakar and Omilion-Hodges, 2018), the horizontal social exchange relationships, such as team member exchange (TMX) in particular, may exert a more direct influence on innovative behavior. More importantly, even though the positive effects of individual-level TMX have been argued in most research (Shih and Wijaya, 2017; Lee, 2020), largely ignoring the fact that TMX is actually embedded within the broader social context of teams (Kim et al., 2021). This omission is not conducive to fully understand the effectiveness of TMX in the majority of enterprises using teams to accomplish complex jobs. Indeed, this deficiency prompted more scholars (Liao et al., 2010; Liu et al., 2011; Farmer et al., 2015) to call for much more studies on TMX within the context of teams.

To answer the appeal above about considering TMX within the team context, We focused on an extension of the TMX concept, relative team-member exchange (RTMX), as a key instantiation of horizontal social exchange relationship, which represents the actual level of one’s own TMX relationships compared to the average TMX within teams (Farmer et al., 2015). Individuals working in a team not only have a sense of belonging to the team but also see themselves as positively unique within the team (Dierdorff et al., 2018). The differentiated nature of social exchanges including TMX has attracted much more attention (Vidyarthi et al., 2010; Tse et al., 2012; Farmer et al., 2015). However, the knowledge of the effectiveness of RTMX is still far from sufficient. To be more specific, we can conclude from the related research that RTMX may have an impact on individuals’ affection. For example, Wu et al. (2018) suggested that what is being exchanged by TMX is mostly socioemotional support, whereas little is learned about the underlying affective mechanisms of RTMX’s influence on innovative behavior. Therefore, this research firstly tried to uncover the mechanisms by employing affective organizational commitment to explain the effects of RTMX on innovative behavior. Our argument is guided by social comparison theory (Festinger, 1954), which suggests that individuals are inclined to use social comparison information to form a self-assessment of their own abilities and guide their work attitudes and behaviors (Wood, 1989). In consequence, we propose that RTMX standing serves to shape individuals’ affective organizational commitment, which, in turn, has a positive impact on innovative behavior.

Another part of our incomplete understanding of RTMX involves how the contexts in which TMX relationships are embedded affect the outputs related to RLMX. Some scholars (e.g., Liu et al., 2011; Kim et al., 2021) have pointed out that TMX may operate at the team level of theory, as TMX differentiation (i.e., “the degree to which the quality of a team member’s exchange relationships with other team members varies”; see Liao et al., 2010, p. 1091), a critical contextual variable surrounding the social comparison process of TMX (Chen and Liu, 2020), creates a team-level context that is important and meaningful to the experience of all team members. Specifically, in each executive team, TMX relationships within teams may be more or less different due to the difference in personality, strengths and majors of members. In teams with low-level TMX differentiation, individuals who are relatively closer to their colleagues may not enjoy the same relative advantages that they might if they were in a team with a higher-level TMX differentiation (Liao et al., 2010). Thus, from a social comparison perspective, we further put forward that the effectiveness of RTMX noted above may be contingent on TMX differentiation at the team level.

**Figure 1:**
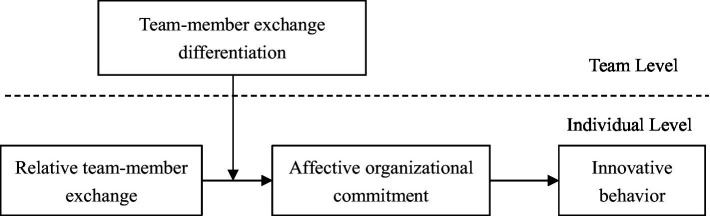
Theoretical model.

Figure 1 depicts our proposed theoretical model. Specifically, in line with recent efforts to expand the taxonomy of TMX research (e.g., TMX differentiation, Liu et al., 2011), we aim to push forward the new field of RTMX in TMX literature in four ways. First of all, we fill the void by employing social comparison theory as an overarching theory for building up a multilevel theoretical framework to examine the impact of RTMX on individual innovative behavior within the context of teams. Second, this study responds to a call by Farmer et al. (2015) to find out underlying mediating processes in associating RTMX with individual outputs. We verify affective organizational commitment as a key psychological mechanism that plays a mediating role in the link between RTMX and innovative behavior. Third, by building up a cross-level moderated mediation model, this study tries to explain the moderating role of TMX differentiation, attempting to probe into why and when RTMX is able to have an impact on affective organizational commitment and, in turn, innovative behavior. The attempt above deepens the understanding of the potential boundary conditions related to the association between RTMX and innovative behavior. Last, the findings of this research provide some useful suggestions for both teams and individuals to deal with the differentiation of TMX relationships within teams.

## Theory and hypotheses

### RTMX and innovative behavior

Relative team-member exchange focuses on differences within teams. Specifically, high and low RTMX offers individuals a reference point to identify their own status relative to other colleagues’ TMX standings. Relative team-member exchange not only can help individuals understand how they define themselves within the teams (Vignoles et al., 2000) but also can bring them some other more valuable resources (e.g., respect, confidence, and trust) through comparisons. We thus believe comparison processes could offer a theoretical framework for explaining the effect of RTMX on individual innovative behavior.

In line with the reasoning above, we contend further that when a member has a high-level RTMX, s/he can be thought to have a positive self-concept (Vignoles et al., 2000; Farmer et al., 2015) and engage in innovative behaviors proactively. Specifically, first, drawing on social comparison theory, members with high RTMX relationships stand at the upper level within the team in terms of their TMX relationships and tend to believe that they can gain much more respect and attention from other members in their teams through downward comparisons (Liao et al., 2010). Thus, these high RTMX members are inclined to perform innovative behaviors so as to maintain a domain position in the team. Second, RTMX provides evaluating information for individuals to understand their outstanding competence (Forsyth, 2000; Farmer et al., 2015). Each member wants to cooperate with capable colleagues to accomplish tasks. High RTMX means that the target individual has a much higher ranking than others within the teams in terms of their TMX score and helps to confirm the abilities of individual members. Therefore, high RTMX members who hold high self-efficacy believe that they have the competence to engage in innovative behavior. Third, when individual members have high-level RTMX, they can gain much more trust from their coworkers (Lau et al., 2021), and then they are likely to enjoy the advantages of collecting much more different and useful information from their colleagues within their teams, thereby behaving innovatively through integrating their own and others’ knowledge and information when they deal with daily tasks (Du et al., 2021).

However, low RTMX individuals may not be as inclined to behave innovatively. Specifically, on the one hand, individual members with low RTMX standings realize that they are at the edge of the group in terms of their TMX rankings through upward comparisons. As they compare their social exchanges with colleagues (i.e., TMX) with other members who hold relatively high TMX, low RTMX members come to realize that they do not get much say within teams and may feel much more uncertain and unworthy when challenging status and thus experience a negative self-concept and decrease the motivation to engage in innovative behaviors. On the other hand, those members with low RTMX become more aware of being detached from their colleagues and getting little assistance and support from others within their teams by making upward comparisons (Farmer et al., 2015). Therefore, they may take action to accomplish the regular work discreetly instead of performing innovative behaviors venturesomely.

Building on the preceding discussion, we believe RTMX could be positively related to individuals’ innovative behavior within their team beyond TMX. Accordingly, we present the following hypothesis:

*Hypothesis 1*: RTMX is positively related to innovative behavior, controlling for individual-level TMX.

### The mediating role of affective organizational commitment

Comparison processes provide a framework for learning how RTMX may have an impact on individuals’ attitudes, including affective organizational commitment. Specifically, on one hand, high RTMX signals the focal member to have closer exchange relationships with other members and facilitates a sense of uniqueness (Farmer et al., 2015). As such, those individuals may realize that others treat them with respect and dignity due to their high-status position within teams, which may contribute to a strong sense of affective organizational commitment. On the other hand, some research has confirmed that individuals may experience more positive feelings when making downward comparisons (Fleischmann et al., 2021), so we tend to contend that individuals who hold high-level RTMX can understand their higher capability and feel more confidence in solving difficulties over others (Stamper and Masterson, 2002), and then generate high-level organizational affective commitment. On the contrary, individuals with relatively lower-level exchange relationships with their colleagues (i.e., low RTMX) are more likely to review themselves as out-group members and then may be affected by negative reciprocity beliefs, so they tend to have weak affective organizational commitment. It is also consistent with those studies showing that perceived outsider status may negatively influence organization commitment (Farmer et al., 2015).

Drawing on social comparison theory, we further emphasize the mediating variable (i.e., affective organizational commitment) that can play in accounting for the effect of RTMX on innovative behavior. Specifically, RTMX serves as salient social comparison information that urges all the members to participate in comparatively estimating their own abilities. This, in turn, helps to form their high-level affective organizational commitment. These affective organizational commitment perceptions that make members much more goal-oriented and proactive directly influence their efforts to engage in innovative behaviors (Yang et al., 2020). Besides, because when individuals have high RTMX, they can gain more trust and respect from their colleagues. This helps to form high affective organizational commitment. These affective organizational commitment perceptions then directly influence innovative behavior. We therefore propose the following hypotheses:

*Hypothesis 2*: RTMX is positively related to affective organizational commitment, controlling for individual-level TMX.

*Hypothesis 3*: Affective organizational commitment mediates the relationship between RTMX and innovative behavior, controlling for individual-level TMX.

### The moderating role of TMX differentiation

As discussed earlier, in a team, a member can form different exchange relationships with peers. Teams with high-level TMX differentiation consist of members who keep social exchange relationships with coworkers that vary widely (Chen and Liu, 2020). Liao et al. (2010) argue further that the degree of TMX differentiation may offer valuable and accurate information to an employee engaging in comparative social evaluation, the reason is that TMX differentiation can be explained as “an indicator of a member’s status in a team.” In consequence, the experience of comparing with colleagues can further influence how an individual reacts to RTMX. In this study, we postulate that TMX differentiation will augment the inflation influence of RTMX in affective organizational commitment from a social comparison perspective.

Specifically, on one hand, high TMX differentiation means team members keep exchange relationship with their coworkers very various (Liao et al., 2010), which signals to members about rich and obvious comparative information. With such high TMX differentiation, as the quality of a member’s social exchanges with colleagues increasing, the member may become increasingly aware that he or she maintains much closer work relationships than do teammates through making downward comparisons (Liao et al., 2010). As a result, the member who enjoys a relatively high TMX relationship within teams tends to realize that he/she is at the center of the team and may be more likely to view him−/herself as more respected and more valued. Then he/she may have a high-level affective organizational commitment and prefer to stay within the organization. On the other hand, TMX differentiation sharpens contrasting perceptions (Ford and Seers, 2006). When TMX differentiation levels are high, some members who keep close exchange relationships with most other colleagues may consider others as “free riders,” while those others may think the former as political operators (Ford and Seers, 2006). In this context, those members who keep high RTMX relationships can clearly realize that they are much more capable and better off than other teammates, it is because they believe that they have taken on more tasks than others. Thus, they hold high affective organizational commitment. On the contrary, as TMX differentiation is at a low level, members may perceive themselves as having a comparable quality of TMX relationships with their teammates (Liao et al., 2010; Liu et al., 2011). In this case, when individual members keep high-level RTMX, they may still regard this relationship to be universal rather than particularly unique or advantageous to themselves and may not think of themselves as in-group members with a higher social position as compared to their colleagues in the team. Thus, the positive effects of RTMX on affective organizational commitment may dwindle. In sum, we postulate that TMX differentiation amplifies the impact of RTMX on affective organizational commitment. Thus, we propose the following hypothesis:

*Hypothesis 4*: TMX differentiation moderates the relationship between RTMX and affective organizational commitment such that RTMX will have a stronger positive effect on affective organizational commitment when TMX differentiation is high rather than TMX differentiation is low.

### Integrated model

To integrate these relationships above, in line with social comparison theory, we propose a multilevel moderated-mediation theoretical model in which TMX differentiation plays a moderating role in the indirect relationship between RTMX and innovative behavior *via* affective organizational commitment. Specifically, when TMX differentiation is high, that is, the quality of exchange relationships between members and colleagues varies greatly (Liao et al., 2010). In this time, individuals with high RTMX who hold a high-status position within teams are inclined to be respected and trusted by more coworkers and feel confident about being better than others by making downward comparisons, so generating high-level affective organizational commitment and, subsequently, innovative behavior. In contrast, when TMX differentiation is low, individuals who have high RTMX cannot feel distinct advantage over others. In this time, RTMX will have a weaker influence on affective organizational commitment and indirectly on innovative behavior. Therefore, we put forward the following hypothesis:

*Hypothesis 5*: TMX differentiation moderates the indirect effect of RTMX on innovative behavior via affective organizational commitment, such that the effects are stronger when TMX differentiation is high rather than TMX differentiation is low.

## Materials and methods

### Sample and procedure

To test the proposed hypotheses, we used a survey-based design to collect data in different organizations located in China. These employees worked in teams and were from different departments, including engineer designing, educational product designing, and software designing. To minimize the potential common method biases, data were collected in a time-lagged design at two-time points. At Time 1 (T1), team members must first report their team-member exchange relationship and affective organizational commitment. At Time 2 (T2), they rated their innovative behavior.

Although the research team made a few attempts to increase the response rate (e.g., sending e-mail reminders and controlling the length of the questionnaires), a few teams and employees did not return their questionnaires. In order to avoid potential random and systematic biases (Allen et al., 2007), teams with within-team response rates higher than 80% were chose for the final sample. The final sample was composed of 51 teams, including 260 team members. 89% response rate for teams and 84% response rate for team members. Among these participants, 54% of the members were female. The average age (in years) was 29.72 for team members. 93% of team members had a bachelor’s degree or higher. The average team tenure (in months) was 35.90, and the average team size was 5.10.

### Measures

According to a back- translation process, our survey questionnaires are translated from English to Chinese. Unless otherwise noted, the measures that the study mentioned were rated employing a 7-point Likert-type scale (1 = *strongly disagree*; 7 = *strongly agree*).

#### Team-member exchange

The 10-item scale that Seers et al. (1995) developed was adopted to measure TMX. A sample item is “I often make suggestions about better work methods to other team members.” Cronbach’s α for this value was 0.89.

#### Relative team-member exchange

Following Farmer et al. (2015), we subtracted the average TMX score of individuals in a team from each team member’s TMX score to evaluate RTMX.

#### TMX differentiation

In line with Liao et al. (2010), we employed the within-team variance in individual-level TMX scores to operationalize TMX differentiation for each team. Much higher within-team variance represents higher-level TMX differentiation (Chen and Liu, 2020).

#### Affective organizational commitment

Consistent with Pundt and Venz (2017), this variable (i.e., affective organizational commitment) was measured using a five-item scale. A sample item is “I would be happy to spend the rest of my career with this organization.” Cronbach’s *α* was 0.89.

#### Innovative behavior

Following Janssen (2000), we captured innovative behavior by using a nine-item measure. A sample item is “transforming innovative ideas into useful applications.” It is worth mentioning that the reasons why we asked individual members instead of team leaders to assess innovative behaviors in this study are as follows. First, employees know more about their own work backgrounds indeed (*cf.* Jones and Nisbett, 1971), so their assessment of the innovative behaviors may be more subtle than those of their leaders. Second, the reporting of innovative behavior is one of the discretionary work behaviors, and very similar to other forms of subjective performance appraisal, raters may vary widely in their assessment of innovative behavior due to their different characteristics (Organ and Konovsky, 1989). Third, leaders are likely to miss genuine employee innovative activities since individual members could only perceive those behaviors intended to impress the leaders (Organ and Konovsky, 1989). Cronbach’s *α* was 0.96.

#### Control variables

In an effort to be consistent with past TMX and innovative behavior research, and to control for the potential influence of individual and group characteristics on the findings of this study, we included several variables as controls. Specifically, at the team level, team tenure was also included as a control variable because it may potentially explain innovative behaviors (Vidyarthi et al., 2010). Accordingly, we also controlled for team size to rule out potential confounds. At the individual level, we controlled for each member’s gender (0 = female, 1 = male) and age (in years) as these variables have been verified to make an impact on the outcome variables in past studies (Wang et al., 2017). Besides, we controlled individuals’ organizational tenure as a control variable. Finally, we included individual-level perceptions of TMX as a control because of its potential influence on both affective organizational commitment and innovative behavior.

## Results

### Descriptive statistics

Before examining the proposed hypotheses, a confirmatory factor analysis of our key individual variables, including TMX, affective organizational commitment, and innovative behavior, was conducted to examine the reliability, convergent validity, and discriminant validity. Both Cronbach’s *α* and composite reliability could be used to assess reliability. All of the Cronbach’s *α* and composite reliability values were greater than the threshold of 0.70, suggesting the reliability of all constructs. To examine the discriminant and convergent validity (Hair et al., 2017), this research conduct a series of confirmatory factor analyses (CFAs). The *χ*^2^, Comparative Fit Index (CFI), Tucker Lewis Index (TLI), Root Mean Square Error of Approximation (RMSEA), Standardized Root Mean Square Residual (SRMR) were employed to test the fit of all models. As shown in Table 1, the three-factor model fits the data better than other models, indicating that our respondents could distinguish the focal constructs clearly. Moreover, the square roots of all of the average variances extracted were larger than the correlations with corresponding other constructs, also indicating an adequate discriminant validity. Besides, all of the average variances extracted were greater than the suggested 0.50, confirming a satisfactory convergent validity.

**Table 1:** Results of confirmatory factor analysis.

Model	*χ* ^2^	*χ*^2^/*df*	Δ*χ*^2^	CFI	TLI	RMSEA	SRMR
3-factor	370.74	1.63	–	0.97	0.96	0.05	0.06
2-factor (TMX + AOC; IB)	740.84	3.10	370.10^**^	0.89	0.88	0.09	0.07
2-factor(AOC + IB; TMX)	1049.17	4.38	678.43^**^	0.83	0.80	0.11	0.13
2-factor(TMX + IB; AOC)	1169.28	4.75	798.54^**^	0.81	0.78	0.12	0.14
1-factor(TMX + AOC + IB)	1817.83	7.24	1447.09^**^	0.67	0.64	0.16	0.15

*N* = 260 for individuals; *N* = 51 for teams. Δ*χ*^2^ tests relative to three factors; TMX, team-member exchange; AOC, affective organizational commitment; IB, innovative behavior; CFI, comparative fit index; TLI, Tucker-Lewis index; RMSEA, root mean square error of approximation; SRMR, standardized root mean square residual.

^**^p < 0.01.

Table 2 showed the means, standard deviations, and correlations among all of the variables. Variables at the individual level are shown in the upper portion of Table 2, and variables at the team level are shown in the lower portion.

**Table 2:** Variable correlations, means, and standard deviations.

Variables	*M*	SD	1	2	3	4	5	6	7
Individual-level variables									
1. Gender	0.46	0.50							
2. Age	29.72	4.94	0.03						
3. Organizational tenure	3.96	3.15	−0.02	0.55^**^					
4. TMX	5.41	0.73	−0.10	0.02	−0.07	**0.71**			
5. RTMX	0.00	0.63	−0.10	0.07	−0.04	0.86^**^			
6. AOC	4.94	1.02	−0.12	0.02	−0.09	0.65^**^	0.64^**^	**0.84**	
7. Innovative behavior	4.81	0.99	0.03	0.01	−0.09	0.52^**^	0.54^**^	0.58^**^	**0.86**
Team-level variables									
1. Team size	5.10	1.79							
2. Team tenure	35.90	34.73	0.35^*^						
3. TMX differentiation	0.62	0.31	0.14	−0.02					

*N* = 260 for individuals; *N* = 51 for teams. *M*, mean; SD, standard deviation; TMX, team-member exchange; RTMX, relative team-member exchange; AOC, affective organizational commitment. The square root values of the average variances extracted are in the main diagonal.

* *p* < 0.05;

** *p* < 0.01.

### Hypotheses testing

Hierarchical linear modeling (HLM) was adopted to test the proposed hypotheses, considering the nested structure of our data and the multilevel nature of these hypotheses.

We first examined null models employing the software HLM 7.0 without any specified predictors to test the significance of between-group variance in the outcomes by examining the significance level of the level-2 residual variance of the intercept (*τ*_00_) and ICC1. The significant results of between-team variance in affective organizational commitment (*τ*_00_ = 0.12, *χ*^2^(50) = 84.16, *p* < 0.01, ICC1 = 0.12), and innovative behavior (*τ*_00_ = 0.13, *χ*^2^(50) = 91.88, *p* < 0.001, ICC1 = 0.14), confirming HLM as the appropriate analytic technique.

We then conducted hierarchical regression analyses with HLM 7.0 by entering control variables and the study variables into different equation steps. Table 3 shows the regression results.

**Table 3:** Results of hierarchical linear modeling analysis for the hypothesized relationships.

	Innovative behavior						Affective organizational commitment			
	M1	M2	M3	M4	M5	M6	M7	M8	M9	M10
Gender	0.11	0.11	0.12	0.11	0.12	0.12	−0.04	−0.03	−0.03	−0.02
Age	−0.00	−0.00	−0.00	−0.00	−0.00	−0.00	0.01	0.01	0.01	0.00
Organizational tenure	−0.02	−0.02	−0.02	−0.02	−0.01	−0.01	−0.01	−0.01	−0.01	−0.01
Team tenure	−0.00	−0.00	−0.00	−0.00	−0.00	−0.00	0.00	0.00^*^	0.00	0.00
Team size	0.02	0.02	0.02	0.02	0.02	0.03	−0.02	−0.02	−0.01	−0.01
TMX	0.79^**^	0.35	0.35	0.29	0.29	0.29	0.98^**^	0.52^**^	0.40	0.40
RTMX		0.49^*^	0.24	0.55^*^	0.43	0.26		0.51^*^	0.62^*^	0.41
TMXD				−0.28	−0.28	−0.28			−0.53^**^	−0.53^**^
RTMX * TMXD					0.40^*^	0.25				0.72^**^
AOC			0.24^*^			0.21^*^				
Deviance	610.35	605.46	595.99	604.25	600.17	593.24	555.72	549.77	545.00	528.12
ΔDeviance		4.89^*^	9.47^*^	1.21	4.08^*^	6.93^*^		5.95^*^	4.77	16.88^**^

*N* = 260 for individuals; *N* = 51 for teams. M, model; RTMX, relative team-member exchange; TMXD, team-member exchange differentiation; AOC, affective organizational commitment. The table shows unstandardized coefficients.

* *p* < 0.05;

** *p* < 0.01.

Hypothesis 1 predicted that RTMX is positively related to innovative behavior, controlling for individual-level TMX. As shown in Model 2 of Table 3, the result indicated that RTMX affected innovative behavior significantly (*β* = 0.49, *p* < 0.05). Therefore, Hypothesis 1 was supported.

Hypothesis 2 proposed that RTMX influences affective organizational commitment positively, controlling for individual-level TMX. The results in Table 3 demonstrated the positive relationship between RTMX and affective organizational commitment (*β* = 0.51, *p* < 0.05). Thus, Hypothesis 2 was supported.

Hypothesis 3 posited that affective organizational commitment plays a mediating role in the relationship between RTMX and innovative behavior, controlling for individual-level TMX. As shown in Model 3 of Table 3, when the mediator (i.e., affective organizational commitment) was entered into the regression model, the positive and significant effect of RTMX on innovative behavior decreased to an insignificant level (*β* = 0.24, *p* > 0.05). Further, we used a parametric bootstrap procedure that employed 20,000 Monte Carlo replications to estimate a confidence interval (CI) around the indirect effect. Results showed that 95% CI was [0.01, 0.26], with zero outside the 95% bias-corrected CI. Therefore, Hypothesis 3 was supported.

**Figure 2:**
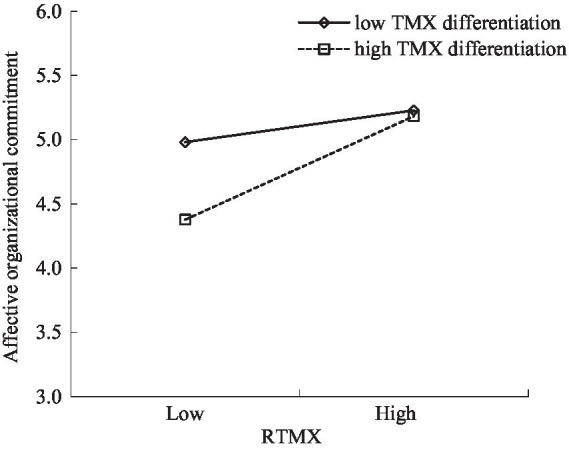
Simple slope of the moderating effect of TMX differentiation on the relationship between relative team-member exchange (RTMX) and affective organizational commitment.

Hypothesis 4 proposed TMX differentiation moderates the relationship between RTMX and affective organizational commitment such that RTMX will have a stronger positive effect on affective organizational commitment when TMX differentiation is high rather than TMX differentiation is low. Results in Model 10 showed that the interaction term of RTMX and TMX differentiation influenced affective organizational commitment positively (*β* = 0.72, *p* < 0.01). Following Aiken and West’s (1991) procedures, we further plotted the interaction at higher and lower levels of TMX differentiation (1SD above and below the mean). As shown in Figure 2, RTMX was more positively related to affective organizational commitment when TMX differentiation was higher rather than when TMX differentiation was lower. Thus, Hypothesis 4 was supported. Moreover, as can be seen from Figure 2, because TMX differentiation may disrupt interpersonal harmony by creating a relational imbalance among team members, which leads to emotional hostility among them (Chen and Liu, 2020), the average level of affective organizational commitment for the group with low TMX differentiation is higher than that of the group with high TMX differentiation. This means that it is necessary to beware of the potential negative effect of TMX differentiation in workgroups.

Hypothesis 5 proposed TMX differentiation moderates the indirect effect of RTMX on innovative behavior *via* affective organizational commitment, such that the effects are stronger when TMX differentiation is high rather than TMX differentiation is low. As shown in Model 6, when the mediator (i.e., affective organizational commitment) was entered into the regression model, the positive and significant influence of the interaction of RTMX and TMX differentiation on innovative behavior decreased to an insignificant level (*β* = 0.25, *p* > 0.05). Further, a Monte Carlo simulation method was applied to obtain 95 percent confidence intervals (CI). Our analysis showed that 95% CI was [0.03, 0.28], with zero outside the 95% bias-corrected CI. Thus, the result proved the moderated mediation models of Hypothesis 5.

## Discussion

In this study, by building up a cross-level moderated mediation model, we tested TMX processes at both the individual and team levels of theory and analysis. Our results examine and support all the hypothesized relationships in the theoretical model. First, we found that RTMX (i.e., TMX relative to a within-group average) has a positive effect on innovative behavior after controlling for individual-level TMX. Moreover, the results showed that affective organizational commitment mediates RTMX and innovative behavior, controlling for individual-level TMX. Finally, we found that TMX differentiation plays a moderating role in the strength of the mediated relationship between RTMX and innovative behavior through affective organizational commitment. These conclusions above offer some significant theoretical contributions to TMX, innovative behavior, and social comparison theory literature and also provide several valuable practical implications for managers and individual members.

### Theoretical implications

The theoretical contributions of this research are threefold. First and foremost, most scholars (e.g., Schermuly and Meyer, 2016; Farh et al., 2017; Lee, 2020) have focused on the effectiveness of TMX at the individual level until now. Considering individual embeds within the broader social context of teams, this research found out the positive linkage between RTMX and innovative behavior. This finding is very consistent with social comparison theory (Festinger, 1954), emphasizing that individuals have self-evaluations and then affect their behaviors by comparing themselves with others. To be specific, RTMX represents the actual level of one’s TMX standing in groups, which offers employees a reference point and context to gain their comparative social evaluations, in turning, leading to individual behavior reactions (i.e., innovative behavior; Greenberg et al., 2007). This finding responds to a call for theoretically and empirically exploring the influence of RTMX (Farmer et al., 2015). Besides, this research deepens the understanding of the impact of RTMX on innovative behavior and further enriches the literature on the construct of RTMX.

Second, even though some scholars have called for more studies to explore mediating processes that might uncover the relationship between RTMX and its outputs, empirical research investigating the indirect influence is still relatively limited. Our paper theoretically and empirically suggested that affective organizational commitment mediates the positive link between RTMX and innovative behavior based upon social comparison theory. In such a case, our findings extend the research of RTMX-innovative behavior linkage by raising a reasonable mediator to understand how RTMX influences innovative behavior.

Third, although individual members inevitably develop different social exchange relationships with their colleagues within the same team (Liao et al., 2010), a very critical limitation so far is the failure to fully realize the moderating role of this horizontal social exchange (i.e., TMX differentiation). Taking a social comparison perspective, we found that TMX differentiation, as a critical boundary condition, moderates the strength of link between RTMX and affective organizational commitment. Furthermore, this research deepens our understanding of the impact of RTMX on innovative behavior using a multilevel moderated-mediation theoretical model. The result showed that TMX differentiation played a moderating role in the strength of the mediated the link between RTMX and innovative behavior through affective organizational commitment. These findings above indicate that TMX relationships do occur at multiple theoretical levels and further emphasize the importance and necessity of taking how the social context created through TMX differentiation affects both individuals’ affection and behaviors into consideration.

### Practical implications

Our study provides the following vital implications to managerial practice. First, our findings suggest that employees with high-level RTMX are more likely to participate in innovative behavior. Thus, to further encourage all members to perform innovative behaviors, it is necessary for managers to help those members with high RTMX realize that they are in a higher insider position and make others also learn about the possibility to develop high RTMX by offering them some opportunities (e.g., training, studying abroad, meetings) to improve their abilities at the same time.

Second, the result shows that RTMX alone is positive for affective organizational commitment, and affective organizational commitment can mediate the direct influence of RTMX on innovative behavior. It is therefore essential for managers to keep all members willing to stay within organizations. For example, one effective way for team leaders is to set clear expectations that members have differentiated strengths that make them unique and valuable contributors to the teams so that they can maintain high-level affective organizational commitment and then do more for the development of their teams (i.e., making an effort in exerting innovative activities).

Last but not least, managers should take the impact of TMX differentiation into consideration, as suggested by the moderating influence that this study identified. Moreover, to further exert the effectiveness of TMX differentiation, team leaders should follow two general guidelines. On the one hand, leaders not only ought to tolerant the existence of TMX differentiation but also should distribute the limited resources fairly, and then make TMX differentiation developed be based upon both ability difference and task allocation. On the other hand, they should take action (e.g., setting up regulations; communicating with subordinates frequently) to avoid potential conflicts and vicious competition caused by TMX differentiation.

### Limitations and directions for future research

Although this research makes several theoretical and applied contributions, some potential limitations still exist. First, the data used to examine our hypotheses came from one cultural background (i.e., China). Therefore, the results may be affected by different cultures and values including power distance, Confucianism, and collectivism (Zhao, 2014). To further determine the generalizability of these new findings, maybe it is necessary for much more scholars to carry out and examine our study again in other cultures. Besides, using survey-based measure to evaluate LMX and TMX may not capture the actual construct of the quality of relationships, thus, we encourage more scholars to measure LMX and TMX adopting different approaches when retesting the proposed hypotheses of our study in future research.

Second, even though this research adopted a time-lagged design and assured the respondents of anonymity to minimize the risks of self-report, it was still very hard to avoid common method biases. It is worth mentioning that some empirical evidence (e.g., Janssen, 2000; Ding and Quan, 2021) supports self-reported innovative behavior, suggesting that self-report may be more subtle than leader-scores. Despite this optimistic observation, we still encourage more scholars to test the proposed hypotheses by employing a multiple-source research design. Specifically, employees can be required to complete measure of TMX, affective organizational commitment, while leaders can rate innovative behavior of each member.

Third, based upon social comparison theory, we explore *how* and *when* RTMX influences innovative behavior only concerning the mediating role of affective organizational commitment and the moderating role of TMX differentiation. Further research can explore other mediating mechanisms (e.g., self-efficacy, network centrality, and psychological ownership) to explore the influence mechanism of RTMX on innovative behavior. Furthermore, other potential moderators (e.g., power distance, team identification, and task complexity) can also be employed from other perspectives. For example, when task complexity is high, instead of paying attention to the intra-group differentiation of TMX, members tend to see RTMX as the result of rational division and then cooperate with each other to accomplish their common jobs, which in turn benefits individual outcomes.

## Conclusion

As noted by Farmer et al. (2015, p. 592), it is necessary to further explore the influence of RTMX. The present study tries to link RTMX to innovative behavior based upon social comparison theory. Specifically, this research indicated that individuals’ within-group TMX (i.e., RTMX) affected innovative behavior positively, and the link above was uncovered to be mediated by affective organizational commitment. Furthermore, TMX differentiation plays a moderating role in the strength of the relationship between RTMX and innovative behavior through affective organizational commitment. All in all, the findings above point out that TMX processes can simultaneously manipulate at multiple theoretical levels to affect innovative behavior within employment relationships.

## Data availability statement

The raw data supporting the conclusions of this article will be made available by the authors, without undue reservation.

## Funding

This research was funded by the Fundamental Research Funds for the Central Universities (B220201049).

## Author contributions

CC and XL contributed to conception and design of the study. CC organized the database, performed the statistical analysis, and wrote the first draft of the manuscript. All authors contributed to the article and approved the submitted version.

## Conflict of interest

The authors declare that the research was conducted in the absence of any commercial or financial relationships that could be construed as a potential conflict of interest.

## Publisher’s note

All claims expressed in this article are solely those of the authors and do not necessarily represent those of their affiliated organizations, or those of the publisher, the editors and the reviewers. Any product that may be evaluated in this article, or claim that may be made by its manufacturer, is not guaranteed or endorsed by the publisher.
